# Hypertension in People with Multiple Sclerosis: Epidemiology, Mechanisms, Hypertensive Disorders of Pregnancy, and Clinical Implications

**DOI:** 10.3390/diseases14100376

**Published:** 2026-10-13

**Authors:** Ahmed Arafa, Amira S. A. Said, Ehab Elkady, Alshimaa Mostafa, Mona S. Ibrahim, Doaa Mahmoud Khalil

**Affiliations:** 1 Department of Public Health, Faculty of Medicine, Beni-Suef University, Beni-Suef 62521, Egypt; doaamahmoud@med.bsu.edu.eg; 2 Department of Clinical Pharmacy, College of Pharmacy, Al Ain University, Abu Dhabi P.O. Box 112612, United Arab Emirates; amira.ahmed@aau.ac.ae; 3 Ada’a Health Center, Ministry of Health, Riyadh 11176, Saudi Arabia; eelkady@moh.gov.sa; 4 Directorate of Health Affairs, Ministry of Health and Population, Menoufia 32951, Egypt; 5 Faculty of Medicine, Beni-Suef University, Beni-Suef 62521, Egypt; alshimaa.mostafa@outlook.com; 6 Department of Obstetrics and Gynecology, Dhurma Hospital, Riyadh 19839, Saudi Arabia; monasi@moh.gov.sa; 7 National Breastfeeding Program, Ministry of Health, Riyadh 11176, Saudi Arabia

**Keywords:** multiple sclerosis, hypertension, blood pressure, hypertensive disorders of pregnancy, pre-eclampsia

## Abstract

Hypertension is a major cardiovascular risk factor, but its epidemiology in people with multiple sclerosis (PwMS) remains unclear. Whether PwMS have a higher prevalence of hypertension than the general population, the potential pathways linking the two conditions, and the association between hypertension and MS-related outcomes remain incompletely understood. This narrative review summarizes current evidence on the prevalence of hypertension in PwMS, examines potential immunological, autonomic, vascular, behavioral, and treatment-related pathways that may link MS and hypertension, and explores how MS disease-modifying therapies may influence blood pressure regulation. It also reviews evidence on the association between hypertension and neurological disability, mobility, cognitive function, visual outcomes, and markers of neurodegeneration in PwMS, as well as hypertensive disorders of pregnancy in women with MS. Finally, it discusses the clinical implications of these findings and identifies priorities for future research.

## 1. Introduction

Multiple sclerosis (MS) is a chronic immune-mediated disorder of the central nervous system (CNS) characterized by inflammatory demyelination, axonal injury, neurodegeneration, and gliosis [1]. People with MS (PwMS) may experience a wide range of neurological and other disabling symptoms, including visual and sensory disturbances, limb weakness, gait impairment, fatigue, mood disorders, chronic pain, sleep disturbance, and cognitive dysfunction [1,2].

The incidence of MS peaks at 30–34 years of age, and women are more frequently affected than men. In 2021, the global prevalence was estimated at 32.3 per 100,000 among women compared with 15.6 per 100,000 among men [3]. Overall, an estimated 1.89 million people worldwide were living with MS in 2021, corresponding to a prevalence of 23.9 per 100,000 population, with more than 62,000 new cases and approximately 16,000 deaths annually [3]. The global prevalence of MS has increased over the past three decades, with the highest burden observed in North America and Western Europe [3]. MS also imposes a heavy economic burden. In the United States (US), the total economic burden of MS was estimated at USD 85.4 billion in 2019, including USD 63.3 billion in direct medical costs and USD 22.1 billion in indirect and nonmedical costs [4].

As survival and long-term management of MS have improved, greater attention has been directed toward comorbid conditions that may affect health and neurological outcomes in PwMS [2,5]. Among these, hypertension warrants particular attention because it is a major modifiable cardiovascular risk factor that can be readily identified and managed in clinical practice [6]. Previous studies have mainly examined individual aspects of the association between MS and hypertension. However, the available evidence has not been comprehensively integrated to clarify the epidemiological association, the potential mechanisms linking the two conditions, the effects of MS treatments on blood pressure (BP), and the possible influence of hypertension on MS-related outcomes. Evidence regarding hypertensive disorders of pregnancy (HDPs) in women with MS is also limited. Therefore, this review aims to summarize the epidemiological evidence on hypertension in MS, discuss the potential immunological, autonomic, vascular, and treatment-related mechanisms linking the two conditions, and examine the association between hypertension and MS-related outcomes. It also reviews the available evidence on HDPs in women with MS and considers the clinical implications and priorities for future research.

## 2. Methods

We conducted a narrative review of the literature on hypertension in PwMS. PubMed and Scopus were searched for relevant articles published up to 1 September 2025, using combinations of terms related to MS, hypertension, BP, and pregnancy (Table S1). We also screened the reference lists of relevant articles to identify additional studies. The identified literature was narratively synthesized according to four main themes: the epidemiology of hypertension in PwMS, potential mechanisms linking MS and hypertension, the association of hypertension with MS-related outcomes, and HDPs in women with MS. This review was not designed as a systematic review, and the selection and synthesis of evidence were conducted using a narrative approach.

## 3. Epidemiological Evidence on the Association Between MS and Hypertension

### 3.1. Epidemiological Evidence

Hypertension prevalence has been evaluated in PwMS relative to people without MS [7,8,9,10,11,12,13,14,15,16,17,18,19,20,21,22] (Table 1). Several large studies using matched populations or adjusted analyses reported a higher prevalence of hypertension among PwMS. The largest study to date used electronic health records from 26 US health networks and included 122,660 PwMS and 37,075,350 individuals without MS. Hypertension was more prevalent among PwMS in both crude (41.5% vs. 22.7%) and adjusted analyses (33.0% vs. 26.4%) [10]. In Taiwan’s National Health Insurance Research Database, a study including 898 PwMS and 4490 age- and sex-matched controls reported hypertension in 16.0% and 12.8%, respectively; the odds ratio (OR) was 1.4 (95% confidence interval [CI]: 1.1, 1.7) [9]. Similarly, an analysis of the UK Clinical Practice Research Datalink involving 12,251 PwMS and 72,572 age- and sex-matched people without MS reported a higher prevalence of hypertension among PwMS (9.7% vs. 7.3%) [16]. In the US Accelerated Cure Project Repository, which included 1548 PwMS and 677 people without MS, the crude prevalence of self-reported hypertension did not differ significantly between the groups (16.1% vs. 17.8%); however, after adjustment for potential confounders, MS was associated with higher odds of hypertension [OR (95% CI) = 1.48 (1.09, 2.00)] [13]. The difference between the crude and adjusted findings further illustrates the importance of accounting for differences in the characteristics of the compared populations.

On the other hand, some studies using matched populations reported either a lower prevalence of hypertension among PwMS or no significant association. A Canadian population-based study including 44,452 PwMS and 220,849 age- and sex-matched people without MS reported a lower incidence of hypertension among PwMS [incidence rate ratio (IRR) (95% CI) = 0.87 (0.75, 0.99)] [17]. Another Canadian study of 4192 PwMS and 20,940 age- and sex-matched controls found a lower prevalence of hypertension among PwMS [20.8% vs. 22.5%; prevalence ratio (PR) (95% CI) = 0.85 (0.80, 0.89)] [21]. Similarly, a US study involving 9949 PwMS and 19,898 age- and sex-matched controls reported a lower hypertension prevalence among PwMS (18.3% vs. 29.3%) [20]. Data from the Canadian Primary Care Sentinel Surveillance System showed little overall difference in hypertension prevalence between PwMS and individuals without MS (22.8% vs. 22.6%), with similar prevalence in women [PR (95% CI) = 1.03 (0.95, 1.12)] and men [PR (95% CI) = 1.02 (0.90, 1.15)] [18].

Studies conducted in selected populations also reflected this inconsistency, although their findings may be less generalizable. Among US male veterans, hypertension was more prevalent among PwMS than among other veterans (46.7% vs. 41.2%) [7]. In contrast, a study of hospitalized adults aged ≥ 65 years found a lower prevalence of hypertension among PwMS than among age- and sex-matched hospitalized controls (7.4% vs. 16.0%) [15]. Other studies based on primary care or clinical cohorts reported a lower prevalence of hypertension among PwMS [14,19,22]. Differences in referral patterns, healthcare utilization, population characteristics, and methods of hypertension ascertainment limit direct comparison of these estimates with those from population-based studies.

Pregnancy-specific studies should also be considered separately because pregnant women represent a younger and clinically distinct population. In California hospital discharge data, chronic hypertension was reported in 2.9% of pregnant women with MS compared with 1.1% of pregnant women without MS [8]. A US claims-based study reported hypertension in 5.6% of women with MS who had live births, compared with 3.5% of women without MS who had live births [11]. In two additional US administrative datasets, pre-existing hypertension was more frequent among pregnant women with MS than among those without MS (9.8% vs. 6.7% in the Truven Health MarketScan database and 3.6% vs. 2.0% in the Nationwide Inpatient Sample) [12].

Overall, the available evidence does not establish that hypertension is consistently more prevalent in PwMS. Some large population-based studies using matched or adjusted comparisons reported a higher prevalence of hypertension, whereas other well-matched studies reported no association or a lower prevalence or incidence. Differences in age, sex, study setting, hypertension ascertainment, comparator selection, and statistical adjustment likely contribute to these inconsistent findings.

### 3.2. Age-Related Patterns

Age may be an important source of heterogeneity in the reported association between MS and hypertension. In the Canadian Primary Care Sentinel Surveillance System, hypertension was more prevalent among PwMS aged 30–39 years than among people without MS (8.1% vs. 5.6%), whereas among those aged ≥ 65 years, hypertension was less prevalent in PwMS (45.3% vs. 55.7%) [18]. A population-based study using administrative data from four Canadian provinces found no clear difference in hypertension incidence between young and middle-aged people with and without MS, but the incidence was approximately 25% lower among PwMS aged ≥ 65 years [17]. Among US male veterans, hypertension was more prevalent overall in PwMS than in controls (46.7% vs. 41.2%), but the difference was small among individuals aged ≥ 50 years (49.9% vs. 48.1%) [7]. In the large analysis of more than 37 million individuals from 26 US health networks, the PR (95% CI) for hypertension comparing PwMS with people without MS was 4.41 (2.87, 6.77) at ages 15–19 years and 2.10 (1.96, 2.24) at ages 30–34 years. The corresponding PR (95% CI) was substantially lower at older ages: 1.27 (1.25, 1.29) at ages 65–69 years and 1.26 (1.24, 1.28) at ages 70–74 years [10]. In another study of 130,050 individuals with hypertension, including 892 PwMS, the mean age at hypertension onset was lower among PwMS than among people without MS (53.3 vs. 60.0 years), although this difference was attenuated after adjustment for potential confounders [23].

Taken together, these findings suggest that differences in hypertension prevalence between people with and without MS may be more apparent at younger ages and diminish with increasing age. One possible explanation is the low background prevalence of hypertension among younger adults, which can produce larger relative differences between groups, whereas the high prevalence of hypertension at older ages may reduce these differences. Other explanations include survival bias, differences in healthcare utilization and medication use, and the increasing contribution of conventional cardiovascular risk factors with age. However, because most studies reported age-stratified estimates rather than formal tests of effect modification, an interaction between age and MS cannot yet be established.

## 4. Potential Mechanisms Linking MS and Hypertension

Several biological, behavioral, and treatment-related pathways may potentially contribute to an association between MS and hypertension. These include shared inflammatory and vascular processes, autonomic and neuroendocrine dysregulation, lifestyle-related factors, and the effects of MS treatments. Most evidence supporting these pathways is indirect, and they should therefore be considered hypothesis-generating rather than established mediators of hypertension in PwMS.

### 4.1. Shared Inflammatory, Endothelial, and Oxidative Pathways

MS and hypertension, although traditionally viewed as disorders of different organ systems, share several pathophysiological features involving chronic inflammation, immune dysregulation, endothelial dysfunction, and oxidative stress.

In MS, failure of immune tolerance leads to activation of autoreactive adaptive and innate immune cells, including CD4^+^ and CD8^+^ T cells, B lymphocytes, and myeloid cells. These cells infiltrate the CNS through a disrupted blood–brain barrier, accumulate in perivascular and parenchymal regions, and create a sustained inflammatory microenvironment. This process is characterized by pro-inflammatory cytokine production, intrathecal antibody responses, and complement activation, resulting in demyelination, axonal damage, synaptic dysfunction, and progressive neurodegeneration [24,25,26]. Similarly, hypertension is increasingly recognized as a chronic immune-mediated vascular disorder rather than a purely hemodynamic condition. Both innate and adaptive immune cells accumulate within the arterial wall, perivascular adipose tissue, and kidneys, where they promote oxidative stress, endothelial dysfunction, arterial stiffness, and maladaptive vascular remodeling through cytokine-driven and neurohumoral mechanisms [27,28].

Another potential mechanistic overlap between MS and hypertension involves shared pro-inflammatory signaling pathways across neural, vascular, and immune systems, including the interleukin-6 (IL-6)–Janus kinase (JAK)–signal transducer and activator of transcription (STAT) axis. In MS, activation of this pathway promotes pathogenic T-cell responses, disrupts blood–brain barrier integrity, and enhances microglial and astrocytic activation, thereby sustaining neuroinflammation [29,30]. In hypertension, IL-6–JAK–STAT signaling contributes to endothelial activation, vascular smooth muscle proliferation, and extracellular matrix remodeling, promoting arterial stiffness and vascular dysfunction [31]. These inflammatory and oxidative changes may impair endothelial NO signaling through reduced eNOS expression or activity and increased scavenging of NO by reactive oxygen species. At the same time, inflammation may increase iNOS-derived NO and reactive nitrogen species such as peroxynitrite. The resulting imbalance contributes to endothelial dysfunction, impaired vasodilation, and increased vascular resistance [31].

Oxidative stress is another shared pathophysiological feature of MS and hypertension at both systemic and cerebral levels. In MS, excessive reactive oxygen species (ROS) production by activated microglia, macrophages, and dysfunctional mitochondria induces lipid peroxidation, DNA damage, and disruption of the neurovascular unit, exacerbating demyelination and neuronal loss [32,33]. In hypertension, ROS generated by vascular smooth muscle and endothelial cells promote lipid peroxidation, mitochondrial dysfunction, and arterial stiffening, thereby contributing to BP elevation and target-organ damage [34,35,36].

Collectively, these shared inflammatory, endothelial, and oxidative pathways provide biological plausibility for a potential association between MS and hypertension. However, direct evidence that these mechanisms account for an increased risk of hypertension in PwMS remains limited.

### 4.2. Autonomic Nervous System Dysfunction

Autonomic dysfunction is frequently reported in MS and may represent one potential pathway linking MS with BP dysregulation. Cardiovascular autonomic control is governed by the central autonomic network, a distributed neural system comprising the insula, anterior cingulate cortex, amygdala, hypothalamus, brainstem nuclei, and spinal autonomic pathways [37]. Demyelinating and neurodegenerative lesions within this network disrupt the integration of baroreflex, chemoreflex, and cardiovagal control, leading to instability in heart rate and BP regulation in PwMS [38].

Clinical and experimental studies consistently demonstrate that PwMS may exhibit reduced heart rate variability (HRV) and baroreflex dysfunction, which are features of cardiovascular autonomic dysfunction [39]. A recent meta-analysis of 43 studies including 1518 PwMS and 1062 healthy controls showed significantly reduced HRV, with lower root mean square of successive differences, percentage of normal-to-normal intervals differing by more than 50 milliseconds, and high-frequency and low-frequency power, indicating parasympathetic withdrawal and sympathetic dominance in MS [40]. Reduced HRV is associated with hypertension [41,42]. Reduced parasympathetic activity and persistent sympathetic overactivation lead to increased heart rate, peripheral vasoconstriction, and renin–angiotensin–aldosterone system activation, which together promote sustained elevations in BP and vascular remodeling [41,42,43].

Physiological evidence of baroreflex dysfunction in MS further supports the biological plausibility of this pathway. Huang et al. demonstrated that people with relapsing–remitting MS exhibit a blunted carotid baroreflex-mediated increase in BP in response to simulated hypotension due to an impaired ability to reduce vascular conductance, despite preserved heart-rate responses [44]. This pattern indicates selective failure of sympathetic vasoconstrictor control rather than normal cardiovascular regulation. Impaired baroreflex buffering can increase BP variability and may therefore contribute to abnormal BP regulation [45].

Furthermore, autonomic dysfunction in MS appears to be biologically linked to disease activity and progression. In a longitudinal study of early-diagnosed PwMS, autonomic symptom burden, measured by the Composite Autonomic Symptom Score, independently predicted the risk of new relapses and MRI disease activity [46]. PwMS with higher autonomic symptom scores had a 2.7-fold higher hazard of relapse, while lower epinephrine levels further increased relapse risk [46]. These findings support an association between autonomic dysfunction and MS disease activity, although they do not establish that autonomic dysfunction contributes to hypertension in PwMS.

MS-associated fatigue, anxiety, and sleep disturbances may also contribute to autonomic and cardiovascular dysregulation. Chronic fatigue and psychological stress increase hypothalamic and limbic system activation, shifting autonomic balance toward sympathetic dominance and reducing cardiovagal tone [47,48,49]. Sleep fragmentation, insomnia, and circadian rhythm disruption, common in MS, further impair nocturnal pressure dipping, baroreflex sensitivity, and HRV, thereby promoting sustained sympathetic activation and vascular stiffness [47,48]. Recurrent nocturnal autonomic arousals and impaired sleep-related BP recovery expose the cardiovascular system to prolonged hemodynamic stress, which can accelerate endothelial dysfunction [49].

### 4.3. Hypothalamic–Pituitary–Adrenal (HPA) Axis Dysregulation

Neuroendocrine dysregulation constitutes a potential pathway linking MS to hypertension risk. Central to this framework is the HPA axis, a core integrative system that orchestrates neuroendocrine stress responses, autonomic outflow, circadian rhythmicity, immune modulation, and cardiovascular homeostasis [50].

In MS, sustained neuroinflammatory activity, cumulative psychological stress, and focal as well as diffuse structural injury involving hypothalamic and limbic circuits may collectively disturb HPA axis integrity, leading to maladaptive hormonal signaling and autonomic imbalance [51]. Neuroimaging studies provide in vivo evidence of hypothalamic involvement in MS. Using proton magnetic resonance spectroscopy, Kantorová et al. demonstrated marked neurochemical alterations within the hypothalamus of patients with relapsing–remitting and secondary progressive MS compared with healthy controls [52]. Notably, elevated hypothalamic glutamate and myo-inositol concentrations, markers of excitotoxic stress and glial activation, were preferentially observed in patients with active disease and were strongly associated with greater neurological disability, fatigue severity, and depressive symptom burden [52]. Complementary findings from early disease stages further reinforce this mechanistic paradigm. Hnilicová et al. reported significant hypothalamic metabolic abnormalities in treatment-naïve patients following a first clinical MS episode, characterized by reduced N-acetyl aspartate-to-creatine ratios and increased glutamate-, myo-inositol-, and choline-related metabolite ratios relative to healthy controls [53]. The presence of these hypothalamic metabolic abnormalities at the earliest stages of MS, including evidence of neuroaxonal dysfunction, glutamate excitotoxicity, and glial activation, suggests that hypothalamic involvement may occur early in the disease course rather than solely as a consequence of advanced MS.

Post-mortem neuropathological evidence further substantiates the involvement of HPA axis dysregulation in MS. In a comprehensive autopsy study, HPA axis activity, indexed by cerebrospinal fluid cortisol concentrations and the density of corticotropin-releasing hormone (CRH)-expressing neurons in the hypothalamus, was closely associated with neurodegeneration, lesion burden, and overall disease severity [54]. Moreover, hypothalamic CRH expression showed a strong positive correlation with cerebrospinal fluid glutamate levels, particularly in primary progressive MS, suggesting that progressive neurodegeneration may act as a potent driver of sustained HPA axis activation [54]. These findings provide direct anatomical and biochemical evidence linking hypothalamic injury, excitatory neurotransmission, and chronic neuroendocrine stress signaling in MS.

Evidence from cardiovascular research has also linked HPA axis dysfunction with hypertension [55]. In a controlled study of unmedicated hypertensive men, Wirtz et al. observed a blunted cortisol awakening response and impaired negative feedback following dexamethasone suppression, indicative of dysregulated HPA axis responsiveness [56]. At the population level, an MRI study involving 400 individuals without known cardiovascular disease demonstrated that adrenal gland volume, a surrogate marker of chronic HPA axis activation, was independently associated with hypertension, left ventricular remodeling index, and increased left ventricular wall thickness [57].

Taken together, these findings suggest that HPA axis dysregulation could represent another biological pathway linking MS and hypertension. However, the available evidence is indirect, and studies specifically examining whether HPA axis abnormalities contribute to hypertension in PwMS are needed.

### 4.4. MS Medications

Pharmacological treatment of MS may influence BP regulation. The available studies, however, have assessed different outcomes, including acute hemodynamic changes, longitudinal changes in measured BP, hypertension reported as a treatment-emergent adverse event, incident hypertension during follow-up, and broader cardiovascular outcomes.

Corticosteroids, particularly high-dose intravenous methylprednisolone used to treat acute relapses, are the most well-established contributors to treatment-related BP elevation [58,59]. Glucocorticoids exert mineralocorticoid activity, leading to sodium and water retention, plasma volume expansion, and increased vascular sensitivity to catecholamines, which can acutely elevate BP [60,61]. Although repeated corticosteroid exposure could potentially contribute to longer-term BP elevation, direct evidence demonstrating sustained or chronic hypertension following corticosteroid treatment in PwMS remains limited.

Beyond corticosteroids, increasing attention has been directed toward disease-modifying therapies (DMTs). Among these agents, sphingosine-1-phosphate receptor (S1PR) modulators, including fingolimod, siponimod, ozanimod, and ponesimod, represent an important class of oral DMTs for relapsing forms of MS. These agents reduce relapse rates and may modestly delay disease progression by selectively modulating S1PR, thereby limiting the egress of autoreactive T and B lymphocytes from secondary lymphoid tissues and attenuating immune-mediated inflammation [62,63,64,65,66,67]. S1PR modulators may influence BP through their effects on cardiovascular and autonomic signaling. These agents primarily target S1PR1, which is expressed not only on lymphocytes but also on atrial myocytes, endothelial cells, and vascular smooth muscle. Initial S1PR1 agonism can alter autonomic balance and vascular tone, while chronic receptor modulation and downregulation may shift S1P signaling toward sustained increases in BP [66,67].

Randomized trials of S1PR modulators have generally reported hypertension as a treatment-emergent adverse event rather than directly demonstrating persistent chronic hypertension. In the placebo-controlled FREEDOMS trial, fingolimod was associated with cardiovascular adverse events, including bradyarrhythmia at treatment initiation and hypertension [68]. Hypertension was also reported as a cardiovascular adverse event in the TRANSFORMS trial comparing oral fingolimod with interferon beta-1a [69]. In a pooled analysis of randomized controlled trials including more than 13,000 PwMS, S1PR modulators were associated with a more than two-fold increased risk of cardiovascular adverse events compared with control therapies. The excess risk was driven primarily by bradyarrhythmia and hypertension: RRs = 2.92 and 2.00, respectively. Subgroup analyses further suggested drug-specific profiles, with ozanimod being predominantly associated with hypertension and siponimod with bradyarrhythmia [70]. These findings support a cardiovascular safety signal but do not, by themselves, establish sustained hypertension after treatment.

Monoclonal antibody therapies, including alemtuzumab, natalizumab, and anti-CD20 agents such as ocrelizumab, have transformed the treatment of MS by enabling highly targeted modulation of immune pathways that drive neuroinflammation and disease progression [71,72,73]. Some studies have examined short-term hemodynamic changes rather than incident hypertension. In a retrospective study involving 30 PwMS treated with alemtuzumab and high-dose methylprednisolone compared with 60 age-, sex-, and disability-matched controls treated with high-dose methylprednisolone alone, an increase in systolic BP exceeding 20% from baseline was observed in 5 (16.7%) PwMS in the alemtuzumab group compared with 3 (5.0%) in the control group [74].

Other observational studies evaluated broader cardiovascular outcomes. A nationwide cohort study from Denmark compared cardiovascular outcomes in PwMS treated with fingolimod or natalizumab and found a significantly higher risk of cardiovascular disease among fingolimod-treated PwMS. The study assessed a composite cardiovascular outcome, although hypertension accounted for 82% of the recorded cardiovascular events [75]. Longitudinal changes in measured BP have also been examined separately from diagnosed hypertension. A retrospective cohort study from Saudi Arabia found that, compared with interferons, anti-CD20 therapies (rituximab and ocrelizumab) were associated with an increase in systolic BP of 4.71 mmHg; fingolimod was associated with increases in systolic and diastolic BP of 9.75 and 2.51 mmHg, respectively; dimethyl fumarate was associated with an increase in systolic BP of 13.58 mmHg; and teriflunomide showed the largest increases, with systolic and diastolic BP rising by 15.24 and 4.7 mmHg, respectively [76]. Because this study assessed changes in measured BP, these findings should be distinguished from studies evaluating newly diagnosed hypertension.

In contrast, population-based cohort studies have examined incident hypertension during follow-up. In a large multiregional population-based study from Canada, several DMTs were associated with an increased risk of hypertension, most notably alemtuzumab [adjusted hazard ratio (aHR) 4.96; 95% CI 1.78, 13.84], fingolimod [aHR 1.73; 95% CI 1.30, 2.31], and teriflunomide [aHR 1.76; 95% CI 1.32, 2.37] [77]. Similarly, in a nationwide Danish cohort study, PwMS treated with teriflunomide had a higher incidence of hypertension than those treated with dimethyl fumarate, with incidence rates of 40.6 versus 13.1 per 1000 person-years [78].

Overall, the available evidence suggests that some MS therapies may influence BP regulation or be associated with hypertension; however, the type and strength of evidence vary substantially across treatments and study designs. Acute treatment-related BP elevation, longitudinal changes in measured BP, hypertension reported as an adverse event, and incident hypertension during follow-up should therefore be interpreted separately. Associations reported in observational studies should also be interpreted cautiously, as they do not necessarily establish a causal effect of individual DMTs and may be influenced by residual confounding.

### 4.5. Sedentary Lifestyle and Sleep Disorders

MS-related fatigue, gait impairment, spasticity, and mood disturbances such as depression may contribute to greater sedentary behavior [79,80,81]. A systematic review and meta-analysis of 11 studies, including 1403 PwMS and 449 controls, showed that PwMS engage in significantly more sedentary behavior than non-MS controls, with consistent evidence across studies of generally good methodological quality [82]. Prolonged sedentary behavior is a well-established risk factor for hypertension, acting through impaired endothelial function, increased arterial stiffness, insulin resistance, and chronic low-grade inflammation [83,84].

Sleep disorders, including insomnia, sleep-disordered breathing, restless legs syndrome, narcolepsy, and circadian rhythm dysregulation, are highly prevalent in PwMS [85,86,87]. These conditions are independently associated with an increased risk of hypertension [88,89], potentially through mechanisms such as sympathetic overactivation, intermittent hypoxia, and hormonal dysregulation [90]. As such, sleep disturbances may represent an important, yet often underrecognized, pathway linking MS to elevated BP.

Together, sedentary behavior and sleep disturbances represent potential behavioral pathways through which MS-related symptoms could contribute to hypertension risk. However, direct evidence that these factors explain differences in hypertension prevalence between people with and without MS remains limited.

## 5. Hypertension and MS-Related Outcomes

Hypertension has been evaluated in relation to disease progression and neurological outcomes in MS [5]. One of the potential mechanisms underlying this association is cerebrovascular dysfunction. Neurovascular coupling (NVC) refers to the dynamic association between neuronal activity and cerebral blood flow, which ensures adequate delivery of oxygen and nutrients to metabolically active brain regions [91]. On the other hand, cerebrovascular reactivity (CVR) reflects the capacity of the brain vasculature to increase blood flow following a vasodilatory stimulus. CVR is an essential property for the blood vessels of the brain to maintain nutrient supplies in the face of changing demand [92]. In MS, NVC and CVR may be impaired in association with inflammation, demyelination, and microvascular damage in both gray and white matter [93,94]. Hypertension may further impair cerebrovascular function by promoting arterial stiffness, endothelial dysfunction, and reduced cerebral autoregulatory capacity [95]. These vascular changes may reduce the brain’s ability to increase blood flow in response to neuronal demand, particularly in periventricular and subcortical regions commonly affected in MS. The resulting cerebral hypoperfusion could potentially contribute to neurodegeneration and impaired cognitive processing [95]. Supporting this mechanism, an in vivo MRI study showed that progressive hypertension initially increased NVC and CVR as a compensatory response, but both became impaired in advanced hypertension. These changes were accompanied by reduced microvascular density and neuronal loss [96].

Growing clinical evidence has examined the association between hypertension and MS-related neurological outcomes. Observational and imaging studies indicate that hypertension in PwMS is consistently associated with worse physical function, greater disability accumulation, and adverse neuropsychiatric outcomes, including impaired mobility, depression, visual dysfunction, and structural brain damage [97,98,99,100,101,102,103,104]. In a retrospective cohort of 2083 relapsing–remitting PwMS from the Knowledge Program of Cleveland Clinic, hypertension was associated with lower walking speed, as measured using the Timed 25-Foot Walk Speed [β (95% CI) = −0.18 (−0.26, −0.09)], worse functioning, as measured using the Performance Scale [β (95% CI) = 0.63 (0.40, 0.87)], and depression, as measured using the Patient Health Questionnaire-9 [β (95% CI) = 0.70 (0.41, 0.98)] [97,98]. A cross-sectional study involving 95 relapsing–remitting PwMS showed that higher systolic BP was linked to bilateral reductions in fractional anisotropy within voxels located in the precuneus as well as the middle and posterior cingulate gyrus, indicating compromised white matter integrity [99]. Additionally, systolic BP was negatively associated with regional gray matter volumes, assessed by Voxel-Based Morphometry, in the posterior orbital gyrus, medial frontal cortex, and subcallosal area on both sides of the brain, suggesting frontal brain atrophy [99]. Dagan et al.’s study involved 2396 PwMS who were followed for 20 years and divided them according to their disability status, using the Expanded Disability Status Scale (EDSS), into EDSS4, EDSS6, and EDSS8. PwMS and hypertension had a significantly higher likelihood of reaching each EDSS milestone compared to PwMS only: 62% vs. 43% for EDSS4, 51% vs. 28% for EDSS6, and 17% vs. 10% for EDSS8 [100]. In a cohort study using data from 8983 PwMS enrolled in the North American Research Committee on Multiple Sclerosis Registry, hypertension was associated with a 29% increase in early gait disability, a 25% increase in unilateral assistance, and a 17% increase in bilateral assistance [101]. Another study using data from the same cohort showed a significant association between hypertension and increased risk of mild and moderate visual disability by 32% and 31%, respectively [102]. Robens et al. reported that PwMS with stage II hypertension were almost three times more likely to have severe ambulatory disability compared to those with normal BP [103]. In a five-year longitudinal study of 194 PwMS, those with hypertension showed a greater percentage change in lateral ventricle volume than those without hypertension (24.5% vs. 14.1%), suggesting an association between hypertension and greater brain structural changes over time [104].

In summary, accumulating mechanistic and clinical evidence supports an association between hypertension and worse neurological and structural outcomes in PwMS. However, the available evidence does not establish that hypertension directly causes MS progression. Individuals with more advanced disability may have a higher burden of cardiovascular risk factors, which could contribute to hypertension independently of MS disease activity. Whether BP control can slow MS-related neurodegeneration or disability progression remains to be determined.

## 6. The Association Between MS and HDPs

Because MS predominantly affects women and is most commonly diagnosed during early adulthood, many women receive their diagnosis during their reproductive years, raising concerns about the potential impact of MS on pregnancy outcomes and maternal health [105]. Among pregnancy-related complications, HDPs are increasingly common and represent a major cause of maternal and perinatal morbidity and mortality worldwide [106]. HDPs encompass chronic hypertension, gestational hypertension, pre-eclampsia, eclampsia, and HELLP syndrome [106].

Several studies examined the association between MS and HDPs (Table 2). In a population-based study involving US women with and without MS (n = 198 and 1584, respectively) who had singleton births, the prevalence of pre-eclampsia was 3.8% in women with MS and 5.0% in women without MS [107]. Based on data from the Nationwide Inpatient Sample of the Healthcare Cost and Utilization Project, which included over 18.8 million pregnant women, 10,055 of whom had MS, no association was found between MS and HDPs: OR (95% CI) = 1.10 (0.90, 1.40) [108]. Fong et al. showed similar pre-eclampsia/eclampsia rates among 1185 pregnant women with MS and 4,422,864 pregnant women without MS from California: 3.5% and 3.1%, respectively [8]. In a study of 2115 women with MS and an equal number of women without MS, HDPs were observed in 13.9% of women with MS and 14.2% of women without MS [11]. MacDonald et al. showed that the prevalence of pre-eclampsia was similar among pregnant women with and without MS in two large administrative databases: the Truven Health Market Scan Commercial Claims and Encounters Database (5.1% and 5.2%) and the Nationwide Inpatient Sample (4.2% each) [12]. Other studies using data from Finland (61 women with MS and 55,537 women without MS), Turkey (43 women with MS and 100 women without MS), and England (181 women with MS and 244,573 women without MS) showed no association between MS and pre-eclampsia: ORs (95% CIs) = 0.41 (0.13, 1.31), 1.57 (0.25, 9.79), and 1.00 (0.60, 1.70), respectively [109,110,111]. In a meta-analysis of eight studies (nine cohorts), MS was not associated with pre-eclampsia: OR (95% CI) = 1.00 (0.80, 1.19), with no heterogeneity across studies [112]. Collectively, current evidence does not demonstrate a significant association between MS and HDPs, although the evidence for some individual HDP outcomes remains limited.

A recent study examined the association in the reverse temporal direction. A retrospective cohort study using real-world data from 144,782 women with pregnancy-induced hypertension and a matched cohort of normotensive pregnant women reported a 66% higher subsequent risk of MS in the hypertension group during 18 years of follow-up [113]. This finding raises the possibility of shared underlying pathways but requires confirmation in further longitudinal studies.

## 7. Clinical Implications

The accumulating evidence showing associations between hypertension and worse neurological outcomes in PwMS underscores the importance of cardiovascular risk management in this population. BP monitoring should be incorporated into the routine care of PwMS, particularly in individuals receiving high-dose corticosteroids or DMTs that have been associated with BP elevation. Patients with additional cardiovascular risk factors, such as obesity, diabetes, smoking, or chronic kidney disease, may require closer monitoring and timely referral for cardiovascular evaluation when hypertension is suspected or poorly controlled. This approach aligns with current hypertension guidelines emphasizing cardiovascular risk assessment and appropriate BP monitoring [114,115].

Early identification and management of hypertension may help reduce vascular complications; however, whether BP control improves neurological outcomes or modifies MS progression remains uncertain [116,117]. A multidisciplinary approach may therefore be beneficial, involving neurologists, internists, primary care physicians, and rehabilitation specialists to ensure accurate diagnosis, appropriate antihypertensive therapy, and monitoring for medication-related effects, particularly during corticosteroid and DMT use. Lifestyle counseling on diet, physical activity, and stress management should also be incorporated into routine MS care [118].

Of note, real-world data from the Canadian Primary Care Sentinel Surveillance System suggest that PwMS may achieve better BP control than the general population, possibly due to higher healthcare utilization or increased provider vigilance [18]. Similar findings were reported in a case–control study using data from the UK Clinical Practice Research Datalink [119]. Subgroups such as men, smokers, individuals with obesity, and residents of rural areas were less likely to achieve therapeutic targets [18], highlighting the importance of closer BP monitoring and management in higher-risk subgroups. In addition, the recent finding of a higher subsequent risk of MS among women with pregnancy-induced hypertension warrants further investigation but is not sufficient to support specific neurological screening recommendations at present [113].

At the health systems level, MS registries and clinical trial protocols could benefit from routinely capturing comorbid hypertension and related measures, including antihypertensive medication use, BP control, and cardiovascular events. Greater consideration of vascular comorbidities in MS research and clinical guidance may help clarify their potential role in long-term neurological outcomes.

## 8. Future Research

Despite the growing evidence on the association between hypertension and MS, several critical gaps still need further investigation. First, most current evidence relies on retrospective or cross-sectional data, which limits the ability to determine causality, and few studies have addressed this association in non-Western populations. Therefore, prospective cohort studies that use standardized definitions of hypertension and MS-related outcomes are necessary, especially in understudied populations. Second, research on the impact of BP control and antihypertensive treatment on MS progression remains scarce. Prospective studies are needed to determine whether optimal BP control influences MS progression, including relapse rates, lesion burden, cognitive function, and disability. If observational evidence supports a beneficial effect, randomized clinical trials evaluating BP management strategies in PwMS could then be considered. Third, potential biological mechanisms linking hypertension and MS, such as chronic inflammation, HPA axis dysregulation, and autonomic dysfunction, should be explored using biomarkers, neuroimaging, and experimental models. Such studies are needed to determine whether these pathways contribute to hypertension in PwMS rather than simply representing mechanisms shared by the two conditions. Finally, further large prospective studies are needed to clarify the association between MS and HDPs.

## 9. Limitations

This review has several limitations. First, because it was designed as a narrative rather than systematic review, study identification and selection were not conducted using systematic-review methods, and publication or selection bias cannot be excluded. Second, substantial heterogeneity across the included studies in population characteristics, hypertension definitions, study settings, comparator selection, and adjustment for cardiovascular confounders limited direct comparisons across studies. Much of the epidemiological evidence relied on retrospective administrative or self-reported data, which may be susceptible to misclassification bias. Third, most available studies were conducted in North America and Europe, limiting the generalizability of the findings to non-Western populations. Fourth, evidence supporting the proposed biological mechanisms was largely indirect, and the available observational data could not establish causal relationships between MS, hypertension, and neurological outcomes.

## 10. Conclusions

Current evidence on the association between MS and hypertension remains inconsistent. While several studies reported a higher prevalence of hypertension among PwMS, particularly at younger ages, others reported no association or a lower prevalence or incidence. Several biological and treatment-related mechanisms may potentially link MS and hypertension, including inflammation, autonomic dysfunction, neuroendocrine dysregulation, endothelial dysfunction, and the effects of some MS therapies. However, the contribution of these mechanisms has not been fully established in clinical studies. Hypertension may be associated with greater disability and adverse neurological or structural brain outcomes in PwMS. Further prospective and mechanistic research is needed to clarify the association between hypertension and MS and to determine whether BP control influences neurological outcomes in PwMS.

## Figures and Tables

**Table 1:** Summary of the studies investigating the association between MS and hypertension.

Study ID	Data Sources	Study Population	Hypertension Diagnosis	Findings
Lavela et al. (2012) [7] US	Male veterans from the congressionally chartered Veterans Service Organization	MS: 1142 (mean age = 60.8 y); non-MS veterans: 31,500 (mean age = 59.1 y); & general population: 68,357 (mean age = 39.5 y)	Self-reported	Higher hypertension prevalence in MS (46.7%) than in the veteran population (41.2%) and the general population (20.9%)
Fong et al. (2018) [8] US	Health discharge data of pregnant women from California	MS: 1185 (28.6% < 30 y); non-MS: 4,422,864 (58.3% < 30 y)	ICD-9	Higher chronic hypertension prevalence in MS pregnancies (2.9% vs. 1.1%). OR (95% CI) = 2.8 (2.0, 3.9)
Kang et al. (2010) [9] Taiwan	General population from the National Health Insurance Research Dataset	MS: 898 (age ≥ 15 y); non-MS: 4490 (age- and sex- matched); & general population: 711,605	ICD-9	Higher hypertension prevalence in MS (16.0%) than in matched controls (12.8%) and the general population (14.2%). OR (95% CI) = 1.4 (1.1, 1.7)
Briggs et al. (2021) [10] US	An electronic health record database representing 26 major health networks	MS: 122,660 (age ≥ 15 y, Female = 50%); non-MS: 37,075,350 (age: any age, female = 50%)	Electronic health records	Higher crude (41.5% vs. 22.7%) and adjusted (33.0% vs. 26.4%) hypertension prevalence in MS. PR (95% CI) = 1.82 (1.81, 1.84)
Houtchens et al. (2018) [11] US	Pregnant women from the IQVIA Real-World Data Adjudicated Claims	MS: 2176 (mean age = 32.5 y); non-MS: 39,377 (mean age = 29.3 y) before propensity-score matching	Medical and pharmacy claims	Higher hypertension prevalence in women with a live birth with MS (5.6% vs. 3.5%)
MacDonald et al. (2019) [12] US	Pregnant women from the Truven Health MarketScan Commercial Claims and Encounters Database and the Nationwide Inpatient Sample	Truven: MS: 1439 (mean age = 32.6 y); non-MS: 1,101,165 (mean age = 30.3 y) Nationwide: MS: 2436 (mean age = 31.5 y); non-MS: 4,184,380 (mean age = 27.6 y)	ICD-9	Higher pre-existing hypertension in MS pregnancies (9.8% vs. 6.7% in the Truven study and 3.6% vs. 2.0% in the Nationwide study)
Saroufim et al. (2018) [13] US	A network of 10 MS clinics from the US Accelerated Cure Project Repository	MS: 1548 (mean age = 46.3 y, Female = 77.7%); non-MS: 677 (mean age = 48.7 y, female = 66.8%)	Self-reported	No significant difference in hypertension prevalence (16.1% vs. 17.8%). The association became significant after adjustment: OR (95% CI) = 1.48 (1.09, 2.00)
Simpson et al. (2014) [14] Scotland	Scottish Primary Care Dataset	MS: 3826 (mean age = 53.4 y, female = 72.3%); non-MS: 1,268,859 (mean age = 51.2 y, female = 51.1%)	Primary care data	Lower hypertension prevalence in MS (15.7% vs. 18.4%). OR (95% CI) = 0.65 (0.59, 0.72)
Fleming & Blake (1994) [15] US	Hospitalized elderly with MS vs. an age- and sex-matched group of hospitalized patients without MS from the US Quality of Care Medicare Provider Analysis and Review	MS: 7176 (age ≥ 65 y, female = 75%); non-MS: 7168 (age- and sex-matched)	Medicare records	Lower hypertension prevalence in MS (7.4% vs. 16.0%)
Palladino et al. (2023) [16] UK	The UK Clinical Practice Research Datalink (CPRD) GOLD	MS: 12,251 (mean age = 44.9 y, female = 69.8%); non-MS: 72,572 (age- and sex-matched)	Primary care data	Higher hypertension prevalence in MS (9.7% vs. 7.3%)
Marrie et al. (2016) [17] Canada	Administrative data from British Columbia Ministry of Health, Manitoba Health Information Privacy Committee, Commission d’Accès à l’Information du Quebec, Health Data Nova Scotia	MS: 44,452 (age ≥ 20 y, female = 71.4%); non-MS: 220,849 (age- and sex- matched)	ICD-9 and ICD-10	Lower annual hypertension incidence in MS (1.2% vs. 1.4% in 1995 and 1.6% vs. 1.8% in 2005). IRR (95% CI) = 0.87 (0.75, 0.99)
Marrie et al. (2020) [18] Canada	Canadian Primary Care Sentinel Surveillance System	MS: 2926 (mean age = 52.8 y, female = 74%); non-MS: 968,873 (mean age = 48.9 y, female = 57.1%)	ICD-9 or antihypertensive medications	No significant difference in hypertension prevalence (22.8% vs. 22.6%). PR (95% CI) = 1.03 (0.95, 1.12) in females and 1.02 (0.90, 1.15) in males
Sternberg et al. (2013) [19] US	Baird MS Center, Buffalo Medical Center	MS: 206 (mean age = 52.8 y, female = 75.7%); non-MS: 142 (mean age = 56.4 y, female = 77.5%)	Clinical assessment	Lower hypertension prevalence in MS (19.5% vs. 33.3%)
Allen et al. (2008) [20] US	Statewide Planning and Research Cooperative System	MS: 9949 (mean age = 56.6 y, female = 72.8%); non-MS: 19,898 (age- and sex- matched)	ICD-9	Lower hypertension prevalence in MS (18.3% vs. 29.3%)
Marrie et al. (2012) [21] Canada	Provincial administrative data	MS: 4192 (mean age = 47.5 y, female = 71.7%); non-MS: 20,940 (age- and sex- matched)	ICD-9 and ICD-10	Lower hypertension prevalence in MS (20.8% vs. 22.5%). PR (95% CI) = 0.85 (0.80, 0.89)
Jadidi et al. (2013) [22] Sweden	Migration and Health Cohort	MS: 7664 (88.4% ≤ 60 y, female = 68.1%); non-MS: 66,214 (88.9% ≤ 60 y, female = 68.9%)	ICD-9 and ICD-10	Lower hypertension prevalence in MS (0.42% vs. 0.68%)

OR: odds ratio; PR: prevalence ratio; CI: confidence interval; IRR: incidence rate ratio.

**Table 2:** Summary of the studies investigating the association between MS and HDPs.

Study ID	Data Sources	Study Population	HDP Domains	Risk Estimate (95% CI)
Fong et al. (2018) [8] US	Health discharge data of pregnant women from California	MS: 1185 (28.6% < 30 y); non-MS: 4,422,864 (58.3% < 30 y)	Pre-eclampsia or eclampsia	OR (95% CI) = 1.10 (0.80, 1.50)
Houtchens et al. (2018) [11] US	Pregnant women from the IQVIA Real-World Data Adjudicated Claims	MS: 2176 (mean age = 32.5 y); non-MS: 39,377 (mean age = 29.3 y) before propensity-score matching	HDP	OR (95% CI) = 0.86 (0.68, 1.10)
MacDonald et al. (2019) [12] US	Pregnant women from the Truven Health Market Scan Commercial Claims and Encounters Database and the Nationwide Inpatient Sample	Truven: MS: 1439 (mean age = 32.6 y); non-MS: 1,101,165 (mean age = 30.3 y) Nationwide: MS: 2436 (mean age = 31.5 y); non-MS: 4,184,380 (mean age = 27.6 y)	Pre-eclampsia	Truven study: RR (95% CI) = 0.98 (0.77, 1.23) Nationwide study: RR (95% CI) = 1.00 (0.80, 1.19)
Mueller et al. (2002) [107] US	Washington State linked birth certificates and hospital discharge records	MS: 198 (36% < 30 y); non-MS: 1584 (66.5% < 30 y)	Pre-eclampsia	RR (95% CI) = 0.70 (0.30, 1.60)
Kelly et al. (2009) [108] US	2003–2006 Nationwide Inpatient Sample of the Healthcare Cost and Utilization Project	MS: 10,055 (mean age = 31.6 y); non-MS: 18,800,000 (mean age = 27.5 y)	Gestational hypertension, pre-eclampsia, eclampsia, and superimposed pre-eclampsia	OR (95% CI) = 1.10 (0.90, 1.40)
Jalkanen et al. (2010) [109] Finland	All neurology and obstetric units from the 15 central hospitals in Finland	MS: 61 (mean age = 31.2 y); non-MS: 55,547 (mean age = 30 y)	Pre-eclampsia	OR (95% CI) = 0.41 (0.13, 1.31)
Yalcin et al. (2017) [110] Turkey	All deliveries within 10 years from a single tertiary center	MS: 43 (mean age = 30.2 y); non-MS: 100 (mean age = 29.7 y)	Pre-eclampsia	OR (95% CI) = 1.57 (0.25, 9.79)
Goldacre et al. (2017) [111] England	The Oxford Record Linkage Study	MS: 181 (88.4% < 35 y); non-MS: 244,573 (92.5% < 35 y)	Pre-eclampsia	OR (95% CI) = 1.00 (0.60, 1.70)

OR: odds ratio; RR: relative risk; CI: confidence interval; HDPs: hypertensive disorders of pregnancy.

## Data Availability

We did not generate primary data.

## Supplementary Materials

The following supporting information can be downloaded at https://www.mdpi.com/article/10.3390/diseases14100376/s1: Table S1: PubMed Search Strategy.
