# Supplementary material for: Hypertension in People with Multiple Sclerosis: Epidemiology, Mechanisms, Hypertensive Disorders of Pregnancy, and Clinical Implications
Source: Diseases. 2026 Oct 13;14(10):376. doi: 10.3390/diseases14100376 (PMC13703383; doi:10.3390/diseases14100376)
Supplement: Supplementary file 1 [file diseases-14-00376-s001.zip › diseases-4541170-supplementary.pdf]

Supplementary Table 1: PubMed search strategy

Search: Multiple sclerosis AND (hypertension OR blood pressure OR pregnancy OR gestational hypertension OR pregnancy-induced hypertension OR preeclampsia OR pre-eclampsia OR eclampsia OR HELLP)<br>("multiple sclerosis"[MeSH Terms] OR ("multiple"[All Fields] AND "sclerosis"[All Fields]) OR "multiple sclerosis"[All Fields]) AND ("hypertense"[All Fields] OR "hypertension"[MeSH Terms] OR "hypertension"[All Fields] OR "hypertension s"[All Fields] OR "hypertensions"[All Fields] OR "hypertensive"[All Fields] OR "hypertensive s"[All Fields] OR "hypertensives"[All Fields] OR ("blood pressure"[MeSH Terms] OR ("blood"[All Fields] AND "pressure"[All Fields]) OR "blood pressure"[All Fields] OR "blood pressure determination"[MeSH Terms] OR ("blood"[All Fields] AND "pressure"[All Fields] AND "determination"[All Fields]) OR "blood pressure determination"[All Fields] OR "arterial pressure"[MeSH Terms] OR ("arterial"[All Fields] AND "pressure"[All Fields]) OR "arterial pressure"[All Fields]) OR ("pregnancy"[MeSH Terms] OR "pregnancy"[All Fields] OR "pregnancies"[All Fields] OR "pregnancy s"[All Fields]) OR ("hypertension, pregnancy induced"[MeSH Terms] OR ("hypertension"[All Fields] AND "pregnancy induced"[All Fields]) OR "pregnancy-induced hypertension"[All Fields] OR ("gestational"[All Fields] AND "hypertension"[All Fields]) OR "gestational hypertension"[All Fields]) OR ("hypertension, pregnancy induced"[MeSH Terms] OR ("hypertension"[All Fields] AND "pregnancy induced"[All Fields]) OR "pregnancy-induced hypertension"[All Fields] OR ("pregnancy"[All Fields] AND "induced"[All Fields] AND "hypertension"[All Fields]) OR "pregnancy induced hypertension"[All Fields]) OR ("pre eclampsia"[MeSH Terms] OR "pre eclampsia"[All Fields] OR "preeclampsia"[All Fields]) OR ("pre eclampsia"[MeSH Terms] OR "pre eclampsia"[All Fields] OR ("pre"[All Fields] AND "eclampsia"[All Fields]) OR "pre eclampsia"[All Fields]) OR ("eclampsia"[MeSH Terms] OR "eclampsia"[All Fields] OR "eclampsias"[All Fields]) OR ("hellp syndrome"[MeSH Terms] OR ("hellp"[All Fields] AND "syndrome"[All Fields]) OR "hellp syndrome"[All Fields] OR "hellp"[All Fields]))<br>**Translations**<br>**Multiple sclerosis:** "multiple sclerosis"[MeSH Terms] OR ("multiple"[All Fields] AND "sclerosis"[All Fields]) OR "multiple sclerosis"[All Fields]<br>**hypertension:** "hypertense"[All Fields] OR "hypertension"[MeSH Terms] OR "hypertension"[All Fields] OR "hypertension's"[All Fields] OR "hypertensions"[All Fields] OR "hypertensive"[All Fields] OR "hypertensive's"[All Fields] OR "hypertensives"[All Fields]<br>**blood pressure:** "blood pressure"[MeSH Terms] OR ("blood"[All Fields] AND "pressure"[All Fields]) OR "blood pressure"[All Fields] OR "blood pressure determination"[MeSH Terms] OR ("blood"[All Fields] AND "pressure"[All Fields] AND "determination"[All Fields]) OR "blood

pressure determination"[All Fields] OR "arterial pressure"[MeSH Terms] OR ("arterial"[All Fields] AND "pressure"[All Fields]) OR "arterial pressure"[All Fields]

pregnancy: "pregnancy"[MeSH Terms] OR "pregnancy"[All Fields] OR "pregnancies"[All Fields] OR "pregnancy's"[All Fields]

gestational hypertension: "hypertension, pregnancy-induced"[MeSH Terms] OR ("hypertension"[All Fields] AND "pregnancy-induced"[All Fields]) OR "pregnancy-induced hypertension"[All Fields] OR ("gestational"[All Fields] AND "hypertension"[All Fields]) OR "gestational hypertension"[All Fields]

pregnancy-induced hypertension: "hypertension, pregnancy-induced"[MeSH Terms] OR ("hypertension"[All Fields] AND "pregnancy-induced"[All Fields]) OR "pregnancy-induced hypertension"[All Fields] OR ("pregnancy"[All Fields] AND "induced"[All Fields] AND "hypertension"[All Fields]) OR "pregnancy induced hypertension"[All Fields]

preeclampsia: "pre-eclampsia"[MeSH Terms] OR "pre-eclampsia"[All Fields] OR "preeclampsia"[All Fields]

pre-eclampsia: "pre-eclampsia"[MeSH Terms] OR "pre-eclampsia"[All Fields] OR ("pre"[All Fields] AND "eclampsia"[All Fields]) OR "pre eclampsia"[All Fields]

eclampsia: "eclampsia"[MeSH Terms] OR "eclampsia"[All Fields] OR "eclampsias"[All Fields]

HELLP: "hellp syndrome"[MeSH Terms] OR ("hellp"[All Fields] AND "syndrome"[All Fields]) OR "hellp syndrome"[All Fields] OR "hellp"[All Fields]
